# A retrospective study differentiating nontuberculous mycobacterial pulmonary disease from pulmonary tuberculosis on computed tomography using radiomics and machine learning algorithms

**DOI:** 10.1080/07853890.2024.2401613

**Published:** 2024-09-16

**Authors:** Lihong Zhou, Yiwen Wang, Wenchao Zhu, Yafang Zhao, Yihang Yu, Qin Hu, Wenke Yu

**Affiliations:** a Zhejiang Tuberculosis Diagnosis and Treatment Center, Zhejiang Chinese and Western Medicine Integrated Hospital, Hangzhou, Zhejiang, China; b Department of Clinical Medical Engineering, The Second Affiliated Hospital, Zhejiang University School of Medicine, Hangzhou, Zhejiang, China; c Department of Radiology, Sir Run Run Shaw Hospital (SRRSH), Zhejiang University School of Medicine, Hangzhou, China; d Zhejiang Chinese Medical University, Hangzhou, Zhejiang, China; e Department of Radiology, Qingchun Hospital of Zhejiang Province, Hangzhou, China

**Keywords:** CT, nontuberculous mycobacteria, pulmonary tuberculosis, radiomics, machine learning

## Abstract

**Objective:**

To evaluate the effectiveness of a machine learning based on computed tomography (CT) radiomics to distinguish nontuberculous mycobacterial pulmonary disease (NTM-PD) from pulmonary tuberculosis (PTB).

**Methods:**

In this retrospective analysis, medical records of 99 individuals afflicted with NTM-PD and 285 individuals with PTB in Zhejiang Chinese and Western Medicine Integrated Hospital were examined. Random numbers generated by a computer were utilized to stratify the study cohort, with 80% designated as the training cohort and 20% as the validation cohort. A total of 2153 radiomics features were extracted using Python (Pyradiomics package) to analyse the CT characteristics of the large disease areas. The identification of significant factors was conducted through the least absolute shrinkage and selection operator (LASSO) regression. The following four supervised learning classifier models were developed: random forest (RF), support vector machine (SVM), logistic regression (LR), and extreme gradient boosting (XGBoost). For assessment and comparison of the predictive performance among these models, receiver-operating characteristic (ROC) curves and the areas under the ROC curves (AUCs) were employed.

**Results:**

The Student’s *t*-test, Levene test, and LASSO algorithm collectively selected 23 optimal features. ROC analysis was then conducted, with the respective AUC values of the XGBoost, LR, SVM, and RF models recorded to be 1, 0.9044, 0.8868, and 0.7982 in the training cohort. In the validation cohort, the respective AUC values of the XGBoost, LR, SVM, and RF models were 0.8358, 0.8085, 0.87739, and 0.7759. The DeLong test results noted the lack of remarkable variation across the models.

**Conclusion:**

The CT radiomics features can help distinguish between NTM-PD and PTB. Among the four classifiers, SVM showed a stable performance in effectively identifying these two diseases.

## Introduction

Nontuberculous mycobacterial pulmonary disease (NTM-PD) refers to a group of infections caused by mycobacteria other than the *Mycobacterium tuberculosis* complex, which causes tuberculosis (TB) [1–3]. NTM are bacteria commonly found in the environment, mainly in soil and water sources. Interestingly, while most people are exposed to NTM daily, only a small proportion of individuals develop NTM-PD. NTM-PD typically affects individuals with certain underlying conditions, such as chronic lung diseases (e.g. bronchiectasis or chronic obstructive pulmonary disease), immune system disorders, or structural abnormalities of the lungs [4,5]. However, in some cases, it can occur in otherwise healthy individuals. Symptoms of NTM-PD may include persistent or chronic cough, shortness of breath, fatigue, weight loss, and occasionally coughing up blood. These symptoms are quite similar to those of other respiratory conditions, such as pulmonary tuberculosis (PTB), making the diagnosis challenging.

Currently, the main methods of distinguishing between NTM-PD and PTB encompass the sputum culture of mycobacterium and the identification of the species. Nevertheless, these methods are resource-intensive, time-consuming, and demand advanced laboratory facilities. Furthermore, the clinical treatment strategies for NTM-PD and PTB are completely different; therefore, early and effective clinical diagnostic methods are needed. Some research has demonstrated that NTM-PD typically exhibits specific changes in computed tomography (CT) imaging [6–9]. Certain imaging features can help with identification, such as cavity formations, parenchymal lesions, tree-in-bud patterns, and bronchiectasis [9–11]. However, these features do not offer adequate and effective markers for discriminating between the NTM-PD and PTB.

Radiomics, an emerging field in medical imaging, focuses on the extraction and analysis of quantitative data from radiographic images [12,13]. It involves the conversion of radiographic images into mineable data, thereby allowing the detection of previously hidden information and patterns. Through the utilization of cutting-edge technologies, including machine learning (ML) and artificial intelligence, radiomics aims to extract relevant features from medical images that can be correlated with patient diagnosis, treatment response, and disease prognosis. Notably, radiomics has demonstrated significant promise in the differentiation and accurate diagnosis of lung diseases, such as pulmonary nodules and early-stage lung cancer [14,15]. Consequently, it offers a potentially reliable approach for distinguishing between NTM-PD and PTB.

In this study, the maximum lesion extraction radiomics was more clinically feasible than representative characterization of lesions extraction radiomics. We expect to select the best-performing ML model to produce results similar to or better than previous studies by using the maximum lesion extraction radiomics.

## Materials and methods

### Patients and datasets

Herein, a retrospective approach was utilized for the collection of data from individuals with NTM-PD or PTB who had undergone non-contrast CT examinations at Zhejiang Chinese and Western Medicine Integrated Hospital (ZJCWMIH) between January 2018 and January 2020. This study was performed in accordance with the Declaration of Helsinki regarding ethical principles for research involving using human samples. The ZJCWMIH institutional review committee granted its approval for this study (2023-YS-139), and informed consent was not required.

All participants had microbiologically confirmed NTM-PD or PTB. Pathogenic microbiological examination was employed as the diagnostic criteria for NTM-PD and PTB. Sputum samples were collected and subjected to bacterial cultures or strain identification. The Löwenstein-Jensen medium was utilized for the growth of Mycobacterium culture. The identification of the NTM species was carried out using the matrix-assisted laser desorption ionization-time of flight mass spectrometry. To be more specific, the diagnosis of NTM-PD adhered to the guidelines provided in the ‘Treatment of Nontuberculous Mycobacterial Pulmonary Disease: An Official ATS/ERS/ESCMID/IDSA Clinical Practice Guideline’ (2020) [4]. Additionally, the diagnosis of PTB followed the criteria stipulated by the National Health Commission of the People’s Republic of China (Diagnostic criteria for pulmonary tuberculosis [WS 288–2017]) [16].

To retain the most relevant data per our research objective, individuals with both diseases, other pulmonary conditions (encompassing neoplasms, interstitial lesions, or infectious diseases), prior thoracic surgical interventions, people living with HIV or compromised CT images due to respiratory motion or metal artefacts, were excluded.

**Figure 1.**
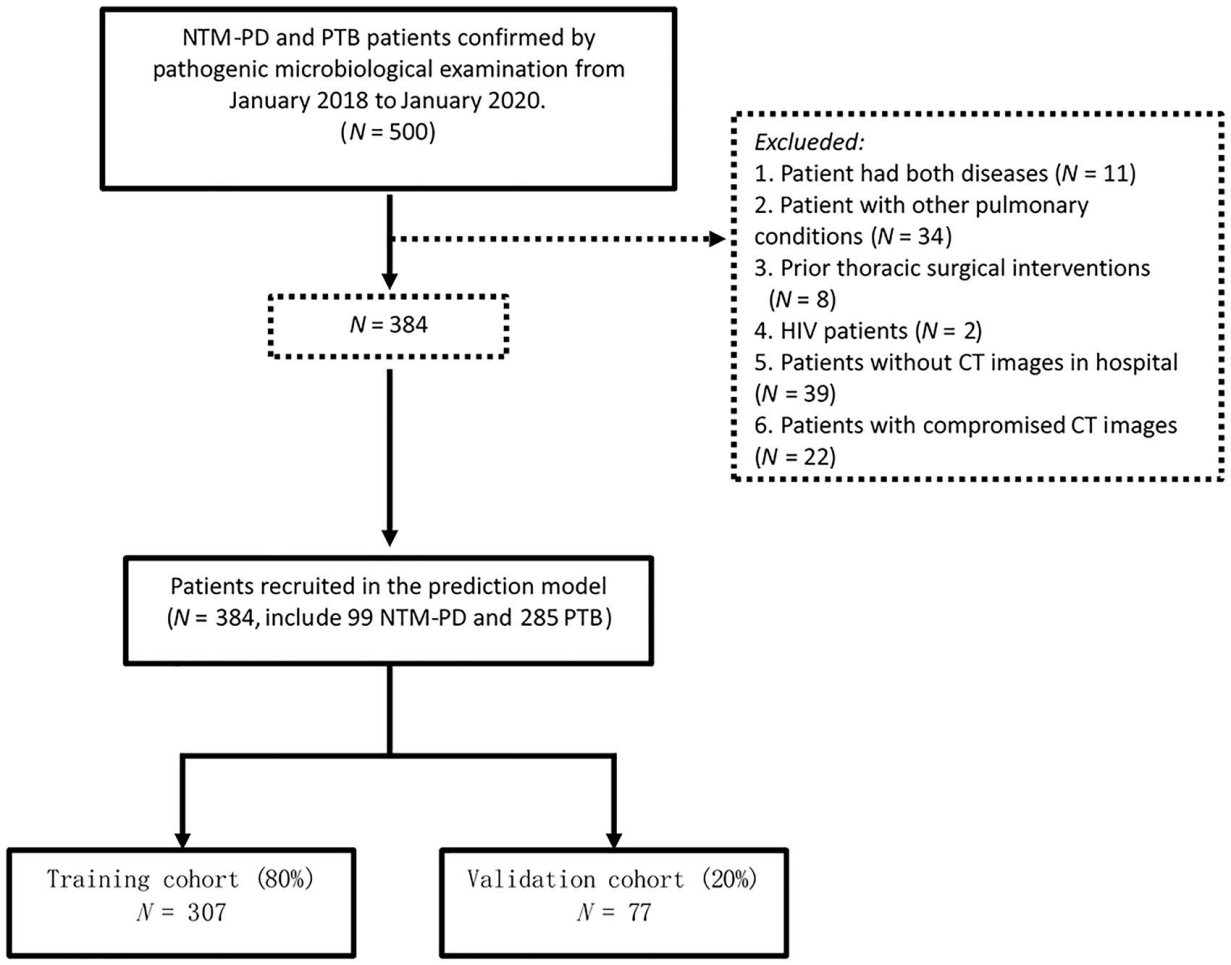
The study flow chart.

This study included 384 patients, comprising 99 individuals with NTM-PD and 285 individuals with PTB. The random categorization of the individuals under study into two cohorts (training and validation) was based on a ratio of 8:2. CT images were obtained within 1 month prior to sample collection for pathogenic microbiological examination, and no clinical treatment was administered during this period (Figure 1).

### Imaging data

CT was performed for all participants using a spiral CT scanner (BrightSpeed, GE, USA) following the same protocol. Throughout the scanning procedure, individuals were positioned in a supine posture and were directed to inhale maximally. They were then asked to hold their breath to ensure data accuracy during the breathing period. A comprehensive scan was performed, covering both lungs from the apical regions to the basal regions. Taking into account the body shape of the individual, further adjustment of the field of view was done. The CT parameters, utilizing a 512 × 512 in-plane size, were configured as mentioned: tube potential, 120 kV; automatic tube current, 0.75 s/r; and collimator width, 16 × 1.25 mm. These scans were reconstructed using both standard and lung algorithms (thickness, 2.5 mm; interval, 2.5 mm). The voxel size of BrightSpeed ranged from 0.31 to 1.14 mm^3^.

### Label annotation

The ITK-SNAP (v 3.8.0, http://www.itksnap.org/) was utilized to label radiographic characteristics, such as cavities, bronchiectasis, nodules, pleural effusion, and consolidation. Two respiratory physicians with 1–3 years of experience (YZ and HY) received a trial-and-error training process to ensure the utmost accuracy. Following their training and the annotation of cases, label verification was carried out by a senior radiologist (WY), who possessed a decade of experience in the field. This verification process was performed without access to clinical information to guarantee consistency. Simultaneously, the senior radiologist compiled a list of any errors or cautions in label annotation, affording the junior physicians an opportunity to address and rectify their mistakes.

### Feature extraction

The area with the largest radiographic characteristics was selected in each patient for extraction of radiomic features [12]. The extraction process was executed *via* Pyradiomics (v 3.6.2), yielding 2153 original features in total [17].

### Feature dimension reduction and ML approach

An 8:2 ratio was utilized for the random categorization of the individuals under study into the training and validation cohorts. Initially, the Student’s *t*-test and Levene test were performed for feature dimension reduction. The least absolute shrinkage and selection operator (LASSO) logistic regression algorithm, which employs a penalty parameter tuned *via* 10-fold cross-validation, was employed to identify the most significant features with non-zero coefficients in the training cohort. Specifically, in the training cohort, the LASSO binary regression model was employed for the selection of 29 (minimum) or 23 (1 standard deviation) radiomic features.

The radiomic features of the training cohort were utilized for the development of four models that were utilized in the training process for the ML algorithms. These models encompassed support vector machine (SVM), random forest (RF), logistic regression (LR), and extreme gradient boosting (XGBoost). The easy-to-operate LR model is frequently used to investigate the effect of trait variables on the target variable, which is typically a binary classifier [18].

As its name suggests, the RF model, designed to mitigate training variation and enhance model generalization and integration, is an ML classifier that employs multiple trees for training and sample prediction [19]. In this research, 240 trees were tuned to achieve the minimum loss rate for 23 radiomic features, whereas 29 radiomic features were represented by 680 trees.

SVM, another widely applied technique in ML algorithms, is a kernel-based approach. It enables the transformation of the feature space with multi-dimensional attributes into two categories [20]. After choosing the linear kernel and setting the cost to 0.0312, gamma to 0.05, and epsilon to 0.45, the best-performing SVM model for the 23 radiomic features was achieved. Meanwhile, the best-performing SVM model for the 29 radiomic features was attained with a cost of 0.25, gamma of 0.05, and epsilon of 0.4.

XGBoost, a state-of-the-art ML algorithm, has been detailed by Chen et al. [21]. It had a more complex parameter configuration. The gbtree booster was selected, where the min_child_weight was set to 0.8, gamma to 0.8, subsample to 1, and colsample_bytree to 1. For the 23 radiomic features, the chosen parameters for optimal performance included an eta of 2.31, nrounds of 29, and max_depth of 5. The optimum parameters for the 29 radiomic features were eta at 1.18, nrounds at 8, and max_depth at 3. All other parameters were kept at the default settings.

### Performance evaluation and statistical analysis

To assess the performance of every model, the receiver-operating characteristic (ROC) curves were generated, and the areas under the ROC curves (AUCs) were computed to assess the predictive power of the models, which was compared using the outcomes of the DeLong test [22].

Baseline features of the individuals were outlined using frequency tables and descriptive statistics. Additionally, the proportions across various infection groups were comparatively assessed through the chi-square (χ^2^) test. LASSO regression was utilized to strike a balance between overfitting and underfitting among the variables. This enabled the identification of the radiomic features that are most crucial for distinguishing between NTM and *M. tuberculosis* lung diseases in this research.

R v 3.6.2 (https://www.r-project.org/) was utilized to perform statistical analyses. The analysis involved the use of the ‘corrplot’, ‘caret’, ‘readr’, ‘randomForest’, ‘glmnet’, ‘pROC’, ‘Matrix’, ‘rms’, ‘e1071’, ‘rmda’, ‘xgboost’, and ‘nsROC’ packages. Additionally, Statistical Product and Service Solutions v 23.0 (IBM) was utilized for certain aspects of the analysis. Two-sided *p* values >0.05 indicated that no remarkable variation existed across the predictive models in terms of their diagnostic performance.

## Results

### Patient characteristics

This study encompassed 99 individuals with NTM-PD and 285 with PTB. Table 1 describes the characteristics of the participants in the training (*n* = 307) and validation cohorts (*n* = 77). During diagnosis, the median age of the participants was calculated to be 52.5 years (interquartile range, 30–65 years). Additionally, the acquired data indicated that there were 153 (39.8%) females among the participants (training cohort, *n* = 125 [40.7%]; validation cohort, *n* = 28 [36.4%]). The distribution of the baseline features across these two cohorts exhibited no significant differences.

**Table 1.** Baseline characteristics of patients with NTM-PD/PTB.

	Training set (*n*, %)	Validation set (*n*, %)	*p* Value
NTM-PD/PTB	307	77	0.804
NTM-PD	80 (26.1)	19 (24.7)	
PTB	227 (73.9)	58 (75.3)	
SEX			0.485
Male	182 (59.3)	49 (63.6)	
Female	125 (40.7)	28 (36.4)	
Age			0.548
Median (IQR)	52 (30–65)	55 (33–65)	

### Clinical radiologic diagnosis

Based on the CT images of the patients with NTM-PD, 15% were correctly diagnosed radiologically and 64% were misdiagnosed with PTB. However, the remaining 21% were diagnosed with pneumonia, bronchiectasis, tumours, and other diseases.

### LASSO regression and feature importance

**Figure 2.**
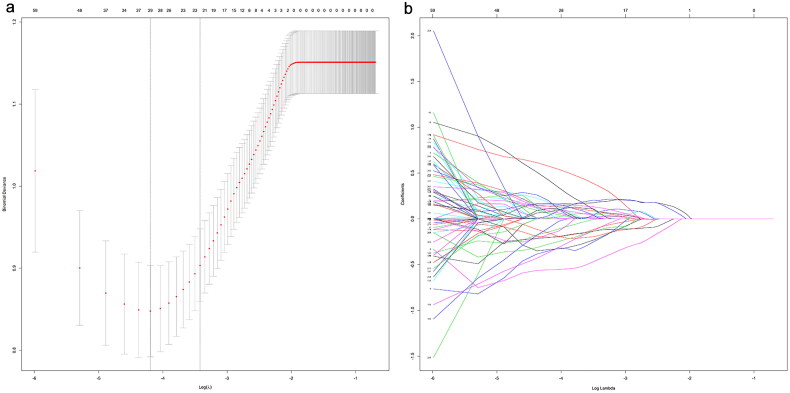
The results of the LASSO regression. The LASSO binary regression model was employed for the selection of 29 (minimum) or 23 (1 standard deviation) radiomics features. LASSO: least absolute shrinkage and selection operator.

**Figure 3.**
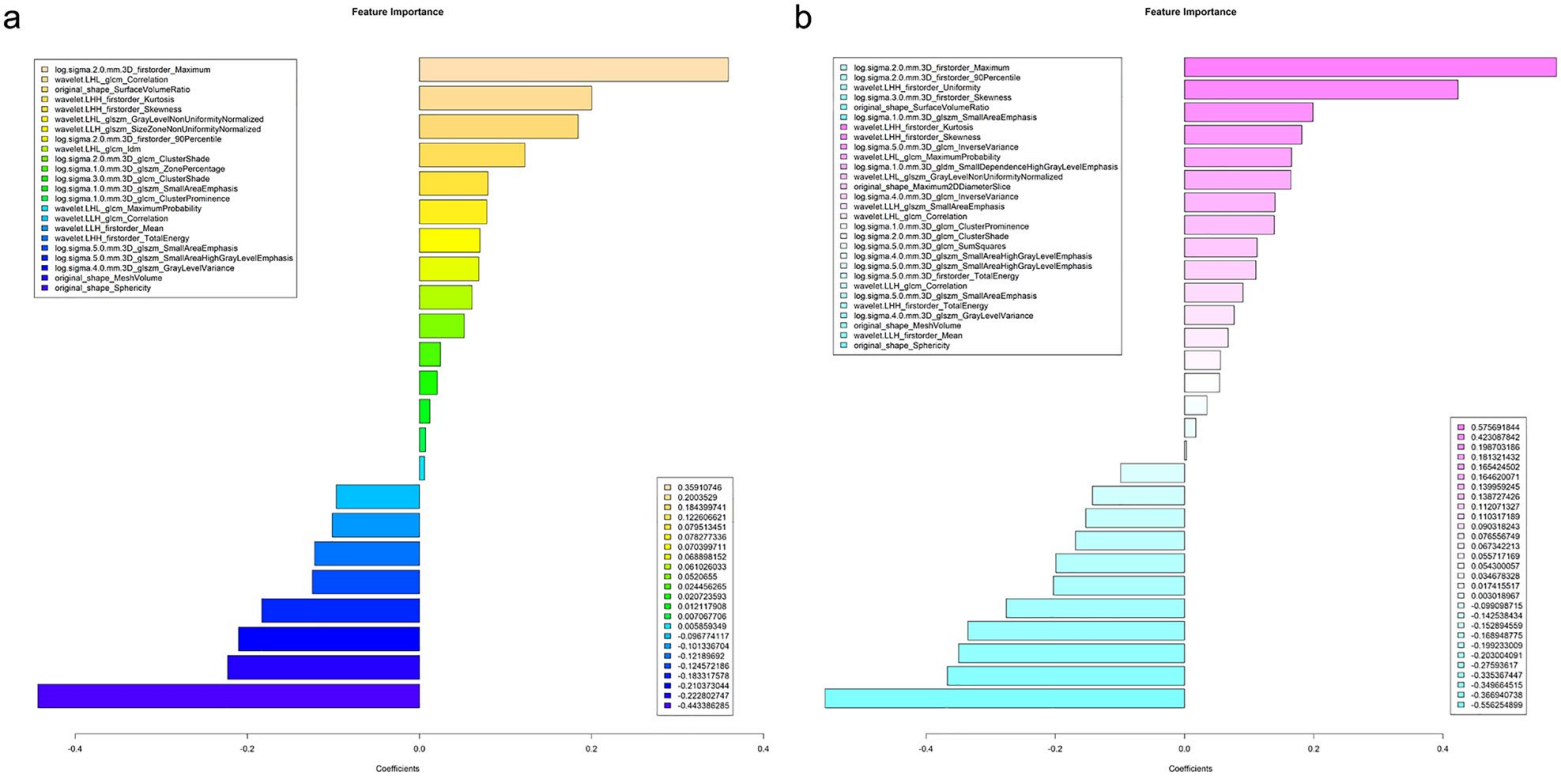
The importance of these selected features was examined through the LASSO regression model. (a) 23 features importance analysis based on LASSO regression. (b) 29 features importance analysis based on LASSO regression.

Overall, 23 or 29 significant radiomics features were identified in the training cohort through the Student’s *t*-test, Levene test, and LASSO LR analysis, all of which had non-zero coefficients (Figure 2a,b). Following this, the importance of these selected features was examined through the LASSO regression model (Figure 3a,b).

### Model evaluation

The four models were assessed *via* ROC curves and their corresponding AUCs to assess their prognostic accuracy. These values were computed for both the training (*n* = 307) and validation cohorts (*n* = 77). The DeLong test suggested the lack of any remarkable variation between the 23 and 29 radiomics features (training cohort, *p* = 0.233; validation cohort, *p* = 0.5298). Therefore, 23 radiomics features were chosen for model establishment.

**Figure 4.**
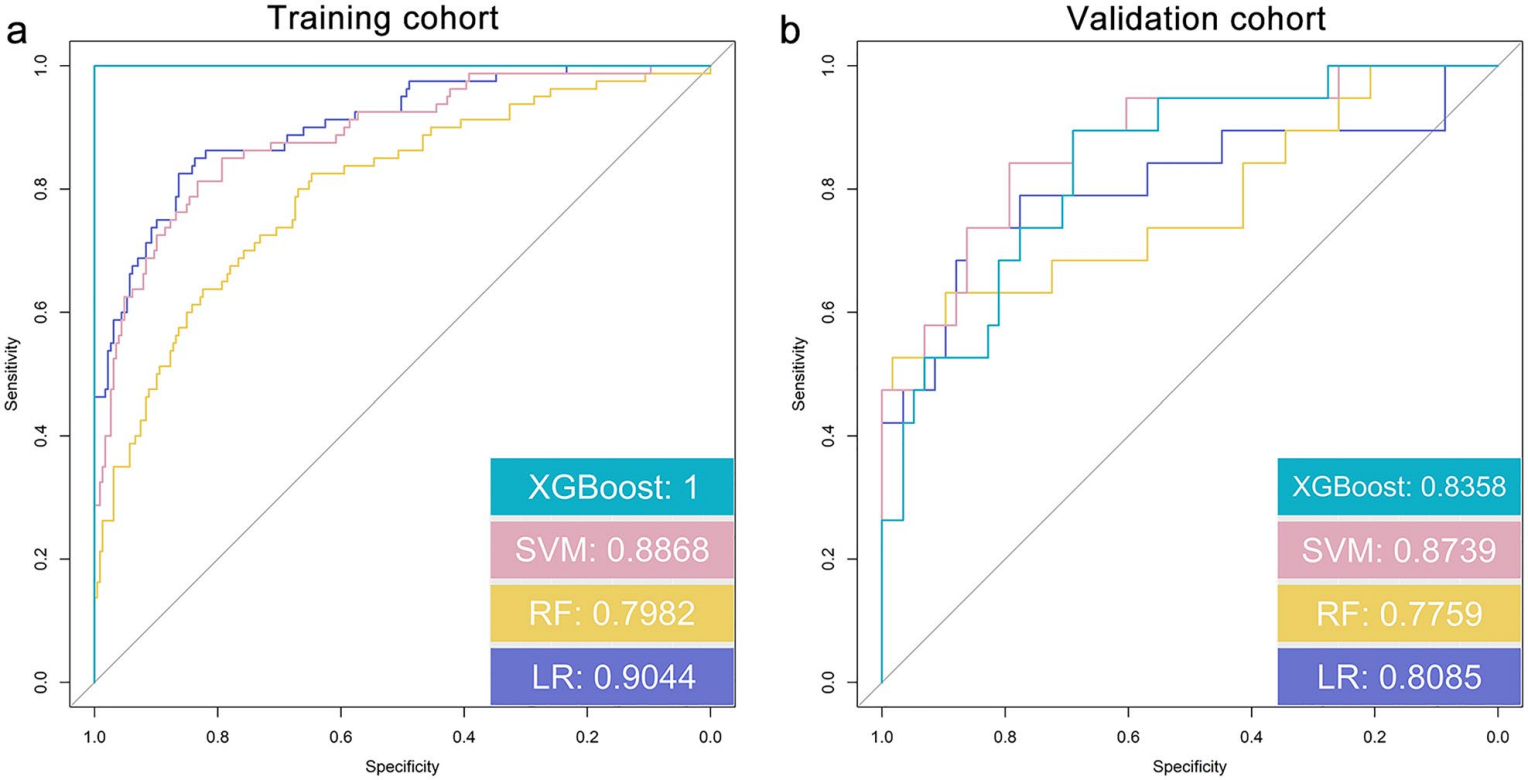
Receiver-operating characteristic curves and AUCs demonstrating the predictions of the four models: LR, RF, SVM, and XGBoost. (a) The training cohort and (b) the validation cohort. AUC: area under the curve; LR: logistic regression; RF: random forest; SVM: support vector machine; XGBoost: extreme gradient boosting.

The four models showed an almost similar performance in the diagnosis prediction. Among these, the XGBoost model outperformed the others in the training cohort (AUC = 1, 95% confidence interval [CI]: 0.8326–0.8474), and was followed by LR (AUC = 0.9044, 95% CI: 0.8150–0.8306), SVM (AUC = 0.8868, 95% CI: 0.8034–0.8202), and RF (AUC = 0.7982, 95% CI: 0.8311–0.8460). As per the data acquired from the DeLong test, the XGBoost model was observed to exhibit statistically significant variation with the other three predictive models (all *p*s < 0.05; Figure 4a).

In the validation cohort, the SVM model exhibited superior accuracy in diagnosis prediction (AUC = 0.87739, 95% CI: 0.8217–0.8513) in comparison to the XGBoost (AUC = 0.8358, 95% CI: 0.7913–0.8249), LR (AUC = 0.8085, 95% CI: 0.8063–0.8372), and RF (AUC = 0.7759, 95% CI: 0.8205–0.8502) models. The data acquired from the DeLong test suggested a lack of remarkable variation among the models (Figure 4b).

## Discussion

NTM-PD refers to lung diseases caused by mycobacteria other than the *M. tuberculosis* complex and *M. leprae*. Previous studies have identified over 190 NTM types, with certain types known to be pathogenic agents [2–5]. It is worth noting that certain regions and countries have seen a rise in the onset and prevalence of NTM-PD [23–25]. Presently, the unique means of identifying NTM involves bacterial culture and strain identification, despite the time-consuming nature of the process. Therefore, early diagnosis and prompt treatment of NTM are of utmost importance.

Nevertheless, distinguishing between NTM-PD and PTB can be challenging due to a significant overlap in symptoms and subtle variations in CT images. Even in specialized hospitals, the accuracy rate of clinical radiologic diagnosis is rather low. This study explores ML to differentiate the individuals with NTM-PD from the PTB ones using CT images. Our research findings revealed that the proposed ML model exhibits significant promise in achieving this distinction.

Previous relevant radiomic studies were based on characteristic radiographic features, such as cavity formation and bronchiectasis [26,27]. Xing et al. used the linear SVM method in the radiomics study of 59 patients with NTM-PD and 57 patients with PTB [26]. The AUCs of cavity formation and bronchiectasis were recorded to be 0.70 ± 0.07 and 0.84 ± 0.06, respectively. Yan et al. used six classifiers (K-Nearest Neighbors [KNN], a machine learning algorithm, SVM, XGBoost, RF, LR, and Decision Tree [DT]) in the radiomics study of lung cavities in 73 NTM-PD and 69 PTB patients, and external verification of 20 NTM-PD and 20 PTB patients [27]. The AUCs of the training group exceeded 0.97, whereas those of the validation group were greater than 0.84, and those of the external validation group were recorded to be higher than 0.84. The ML algorithms have shown good performance in identifying these two diseases. However, the clinical incidence of characteristic radiographic features is not high, and the regional difference is quite large. Kang et al. recorded 421 cases of NTM-PD, and the incidence of cavitation was 21.9% [28]. A review by Hu et al. of 154 patients with NTM-PD in Nanjing, China, showed that the incidence of bronchiectasis was approximately 39.1% [29]. Additionally, Lou et al. reviewed 513 patients with NTM-PD in Shanghai Chest Hospital and found that the incidence rates of bronchiectasis and cavitation in patients with different NTM sub-bacteria were 34.5%–84.1% and 39.1%–85.7%, respectively [30]. Therefore, it seemed more practical for us to select the region with the largest characteristic radiographic features in each patient for radiomics feature extraction.

Ensuring the repeatability and reproducibility of radiomics features in related research can be quite challenging. It is worth noting that the aforementioned studies used a slice thickness of 5 mm, whereas this research used a much thinner slice of 2.5 mm [26,27]. The reconstruction slice thickness has a remarkable influence on the radiomics features, making a thinner slice thickness a more stable option for achieving stable results [31]. Some studies revealed that LOG and Original radiomics features have good stability, whereas Wavelet radiomics features have relatively poor stability [32]. The 23 or 29 radiomics features screened out in our study showed more stable features than those reported in previous studies. Nevertheless, large-sample studies and further research are warranted to confirm these findings.

An additional crucial consideration pertains to selecting the most suitable model. Yan et al. believed that the LR algorithm model is better due to its high precision, recall, and F1 score [27]. Although the XGBoost model outperformed the rest of the models in the training cohort in our study, its performance in the validation cohort was quite ordinary. This could be attributed to XGBoost model overfitting. We agree with Xing et al. that linear SVM may be a better algorithm choice based on its balanced performance in the training and validation cohorts [26]. The deep-learning model has a broader application prospect. However, according to the results by Wang Li et al. and Ying Chiqing et al., the single evaluation index of AUCs was no better than the ML models [33,34]. Therefore, ML algorithms have a role to play, especially when the sample size is relatively small.

## Limitations and future improvement

This study is limited in certain respects. First, the sample size was relatively small, and we were unable to acquire a sufficient number of NTM-PD cases. Second, the study data was from a single centre and lacked real external validation. Third, the research solely relied on CT images, without considering the clinical characteristics, thereby potentially restricting the predictive capability of the ML algorithms. Fourth, the underlying theories of various ML algorithms can be complex and challenging to comprehend. To make these models more accessible for clinicians, not only should they be comprehensible but should also have the capability to estimate their uncertainties [35].

## Conclusion

In this research, 23 and 29 radiomics features were selected based on the LASSO regression model. No remarkable variation was observed between the models of the 23 and 29 radiomics features. Four predictive models were developed to examine the accuracy of distinguishing between NTM-PD and PTB. Considering previous studies and our research, the SVM model may have a stable prediction accuracy according to the AUC analyses as compared to the other algorithms. The radiomics features derived from the CT images proved to be highly effective in distinguishing between NTM-PD and PTB. Notably, the radiomics analysis yielded a more accurate diagnosis compared to assessments made by radiologists.

## Data Availability

The data that support the findings of this study are available from the corresponding author, [WY], upon reasonable request.
